# Gene behaviors-based network enrichment analysis and its application to reveal immune disease pathways enriched with COVID-19 severity-specific gene networks

**DOI:** 10.1093/bioinformatics/btaf378

**Published:** 2025-06-28

**Authors:** Heewon Park, Seiya Imoto, Satory Miyano

**Affiliations:** School of Mathematics, Statistics and Data Science, Sungshin Women’s University, Seoul, Korea; Data Science Center, Sungshin Women’s University, Seoul, Korea; M&D Data Science Center, Institute of Integrated Research, Institute of Science Tokyo, Tokyo, Japan; Human Genome Center, Institute of Medical Science, University of Tokyo, Tokyo, Japan; Human Genome Center, Institute of Medical Science, University of Tokyo, Tokyo, Japan; M&D Data Science Center, Institute of Integrated Research, Institute of Science Tokyo, Tokyo, Japan; Human Genome Center, Institute of Medical Science, University of Tokyo, Tokyo, Japan

## Abstract

**Motivation:**

Gene network analysis is essential for understanding the complex mechanisms underlying diseases, which often involve disruptions in molecular networks rather than individual genes. Despite the availability of large-scale omics datasets and computational tools for gene network analysis, interpretation of the biological relevance of these extensive networks remains challenging.

**Results:**

We propose a novel computational strategy, gene behaviors-based network enrichment analysis, which systematically identifies functional pathways enriched in phenotype-specific gene networks. Our novel method incorporates comprehensive network characteristics, i.e. gene expression levels, edge strengths, and structural patterns of edges, to rank genes based on activity and assess pathway enrichment, effectively identifying functional pathways enriched within these networks. Through simulation studies, our strategy demonstrated superior performance compared with that of existing methods in identifying enriched pathways. We applied this strategy to whole-blood RNA-seq data from 1102 COVID-19 samples provided by the Japan COVID-19 Task Force. The analysis revealed immune disease pathways enriched with COVID-19 severity-specific gene networks, including “Systemic lupus erythematosus” in asymptomatic and severe samples and “Inflammatory bowel disease,” “Primary immunodeficiency,” and “Rheumatoid arthritis” in mild samples. Key biomarkers of COVID-19, such as CXCL8, S100A9, and HLA class I genes, have been identified as critical hub genes and the main players within these networks.

**Availability and implementation:**

Code is available in Figshare (https://doi.org/10.6084/m9.figshare.29093648.v3).

## 1 Introduction

In recent years, gene networks have attracted a large amount of attention in various fields of research, such as statistics, biomedical science, to understand disease mechanisms. Various computational and statistical approaches have been developed to estimate gene networks using large-scale datasets. A gene network consists of more than 10 000 genes and a large number of edges; thus, their interpretation and annotation are challenging tasks. Although many studies have estimated heterogeneous genetic networks, systematically interpreting the biological significance of large-scale gene networks remains challenging.

On the other hand, extensive research is currently underway to understand the function of individual genes and to construct functional knowledge databases such as Gene Ontology and the Kyoto Encyclopedia of Genes and Genomes (KEGG). Furthermore, various computational approaches have been developed to interpret the biological significance of gene sets. Over-representation analysis (ORA) is a commonly used approach for pathway analysis of query gene sets (da Huang *et al.* 2009). ORA determines whether genes associated with known biological functions are over-represented in a query gene set based on an hypergeometric test. Gene set enrichment analyses (GSEA) are also widely used to uncover functional pathways of query gene sets (Subramanian *et al.* 2005). GSEA evaluates the tendency of genes belonging to a functional set to occupy positions at the top or bottom of the gene list, ranked in order of differential expression between phenotypes. Moreover, network-based GSEA, that is, the efficient network enrichment analysis test (NEAT), has been developed (Signorelli *et al.* 2016). NEAT measures the level of enrichment based on the association between genes in the query gene set and those in the functional set. Chang *et al.* (2024) developed a gene set correlation enrichment analysis to examine whether a list of differentially expressed genes is associated with a functional gene set based on a coexpression network. Although various computational approaches have been developed and applied to interpret the biological meaning of a gene set, individual gene-based analysis is not sufficient to understand the complex mechanisms of diseases because they are associated with perturbations in complex molecular networks.

**Figure 1.**
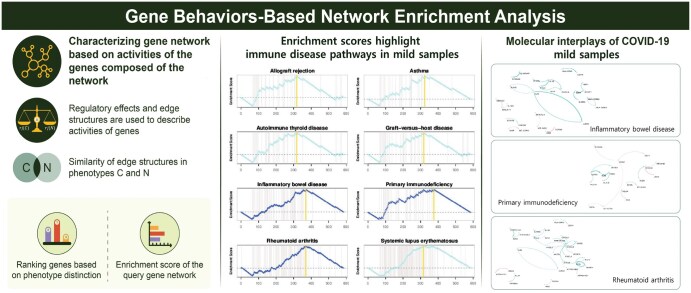
Schematic overview of the GbNEA.

In this study, we aimed to systematically interpret phenotype-specific gene networks and develop a novel computational strategy to reveal functional pathways enriched in phenotype-specific gene networks, called gene behaviors-based network enrichment analysis (GbNEA), based on regulatory effects and Jaccard distances. Figure 1 shows schematic of the GbNEA. Our strategy measured the enrichment of a network based on comprehensive gene network information; our method compares the molecular interplay under two phenotypes based on various characteristics of a gene’s activities and behavior, that is, the expression levels of genes, edge strengths, and edge structures. Then, we ranked the genes according to their activities in the network and tested whether the genes belonging to a functional pathway were over-represented at the top or bottom of the gene ranking list. Incorporating comprehensive information on gene networks leads to effective gene network enrichment analysis and provides biologically interpretable results.

We illustrated the performance of the proposed strategy using simulation studies. The experimental results showed that our strategy outperformed other methods in identifying functional pathways enriched in phenotype-specific gene networks. We also applied our strategy to whole-blood RNA-seq data from 1102 genotyped samples obtained from the Japan COVID-19 Task Force (Wang *et al.* 2022b) and revealed immune disease pathways enriched with COVID-19 severity-specific gene networks (i.e. gene networks of asymptomatic, mild, severe, and most severe samples). Our analysis revealed that the gene networks of COVID-19 asymptomatic and severe samples were enriched in “Systemic lupus erythematosus” pathway, whereas the network of mild samples was enriched in various immune disease pathways, such “Inflammatory bowel disease,” “Primary immunodeficiency,” and “Rheumatoid arthritis.” Our results also identified biomarkers of COVID-19, namely, CXCL8 and S100A9, as hub genes, as well as HLA class I, as the main players in the molecular interplay of COVID-19 severity-specific gene networks. Based on our results and those of scientific literature, we suggest that controlling the identified markers and their interplay may provide crucial clues for understanding the role of the immune system in COVID-19 severity.

## 2 Materials and methods

### 2.1 Gene network estimation

In this study, we considered a directed gene network that describes the causal relationships between genes, where the edges are represented by directed arrows. To describe the causal connectivity between genes, we considered the following linear regression model:


(1) $$y_{i\ell }=\beta_{\ell }^{T}x_{i}+\epsilon_{i\ell },\;i=1,\ldots ,n,\;\ell =1,\ldots ,q,$$

where *n* is the number of samples, *q* is the number of target genes, $y_{\ell }={\left(y_{1\ell },\ldots ,y_{n\ell }\right)}^{T}\in R^{n}$ and $x_{i}={\left(x_{i1},\ldots ,x_{ip}\right)}^{T}\in R^{p}$ are the expression levels of the $\ell^{\text{th}}$ target and *p* regulator genes, respectively. $\epsilon_{i\ell }$ is a random error in the model of the $\ell^{\text{th}}$ target gene. The strength of the directed arrows is estimated using the regression coefficient vector $\beta_{\ell }={\left(\beta_{\ell 1},\ldots ,\beta_{\ell p}\right)}^{T}$, which represents the strength of effect of *p* regulator genes on the $\ell^{\text{th}}$ target gene. That is, the strength of effect from the $j^{\text{th}}$ regulator gene $x_{ij}$ to the $\ell^{\text{th}}$ target gene $y_{i\ell }$ is estimated by ${\hat{\beta }}_{\ell j}$. We consider the following elastic net (Zou and Hastie 2005) to estimate the gene network in the linear regression frame work (i.e. ${\hat{\beta }}_{\ell }$):


(2) $$\hat{\beta_{\ell }}=\underset{\beta_{\ell }}{\arg\min}\{\frac{1}{2}\sum_{i=1}^{n}{\left(y_{i\ell }-\beta_{\ell }^{T}x_{i}\right)}^{2}+\lambda \sum_{j=1}^{p}[\frac{1}{2}(1-\delta )\beta_{\ell j}^{2}+\delta |\beta_{\ell j}|]\},$$

where λ>0 is a regularization parameter that controls the degree of shrinkage for $\beta_{\ell }$ and 0≤δ≤1 is a mixing parameter between the $L_{2}$-norm (i.e. ridge, Hoerl and Kennard 1970) and $L_{1}$-norm (i.e. lasso Tibshirani 1996) penalties.

Although we used a linear regression model with the $L_{1}$-type regularization method to estimate the gene network, our strategy is applicable to any directed gene network estimated by various models and methods.

### 2.2 Gene network enrichment analysis

The aim of this study was to identify the biological pathways enriched with phenotype-specific gene networks. We consider two phenotypes, *C* and *N*. To effectively identify pathways enriched with the query network, we extended the GSEA (Subramanian *et al.* 2005) to network analysis and developed a novel computational strategy called GbNEA based on regulatory effects and Jaccard distance (GbNEA). Our strategy measures network enrichment for a specific pathway based on comprehensive information on the gene network (i.e. expression levels of genes, edge strength, and edge structure). This led to an effective gene network enrichment analysis and biologically interpretable results.

#### 2.2.1 Enrichment score computation

In gene network enrichment analysis, the enrichment score is computed based on the characteristic features of the networks and their rankings. Thus, the characterization and ranking of features are crucial for functional enrichment analysis. In our study, the nodes (i.e. genes) were considered as features that characterize gene networks, and regulatory effects and edge structures were used to describe the activities of genes within gene networks.

1. Characterization of a gene network based on the activities of genes within the network
  1. **Regulatory effects**: The regulatory effects of the $j^{\text{th}}$ regulator genes in the gene network are estimated as (Park *et al.* 2023):
    (3) $$r_{j}^{C}=\sum_{\ell =1}^{q}|{\hat{\beta }}_{C\ell j}{\bar{x}}_{Cj}|\text{and}r_{j}^{N}=\sum_{\ell =1}^{q}|{\hat{\beta }}_{N\ell j}{\bar{x}}_{Nj}|,$$
    where ${\hat{\beta }}_{C\ell j}({\hat{\beta }}_{N\ell j})$ and ${\bar{x}}_{Cj}({\bar{x}}_{Nj})$ are the estimated edge weight from the $j^{\text{th}}$ regulator gene to the $\ell^{\text{th}}$ target gene and the average of expression levels of the $j^{\text{th}}$ gene for the phenotype C(N), respectively. The regulatory effects $r_{j}^{C}$ and $r_{j}^{N}$ measure the strength of the effect of the $j^{\text{th}}$ gene on the gene networks of phenotypes *C* and *N*, respectively.
  2. **Edge structure of the**  $j^{\text{th}}$  **genes**: We consider edge structures as another characteristic of the gene network, because the genes linked in a network may have similar biological functions.
    The degree of the phenotype distinction of edge structure between phenotype *C* and *N* is measured by the following Jaccard distance (Li *et al.* 2014):
    (4) $$dJI_{j}=1-\frac{\left|N_{j}^{C}\cap N_{j}^{N}\right|}{\left|N_{j}^{C}\cup N_{j}^{N}\right|},$$
    where $0\leq dJI_{j}\leq 1$, $N_{j}^{C}$ and $N_{j}^{N}$ are the sets of nodes that are directly connected to the $j^{\text{th}}$ gene in the two networks of phenotypes *C* and *N*, respectively. The small value of $dJI_{j}$ indicates that the $j^{\text{th}}$ gene has many common edges in the networks of phenotypes *C* and *N*, while the $j^{\text{th}}$ gene that shows phenotype-specific molecular interplays has a large value of $dJI_{j}$.
  3. **Characteristics of the phenotype-specific gene network**: To measure degree of the phenotype distinction of the $j^{\text{th}}$ gene, we first consider difference of regulatory effect between phenotypes and propose the following statistic:
    (5) $$d_{j}^{\left(1\right)}(C,N)=r_{j}^{C}-r_{j}^{N},$$
    where $d_{j}^{\left(1\right)}(C,N)$ indicates the degree of difference of the $j^{\text{th}}$ gene’s activities between the networks of the phenotypes *C* and *N*. In order to incorporate not only magnitudes but also sign of edges into the measuring difference of regulatory effect, we also propose the following statistics:
    (6) $$d_{j}^{\left(2\right)}(C,N)=\sum_{\ell =1}^{q}{\left({\hat{\beta }}_{C\ell j}{\bar{x}}_{Cj}-{\hat{\beta }}_{N\ell j}{\bar{x}}_{Nj}\right)}^{2}.$$
    The large absolute value of $d_{j}^{\left(t\right)}(C,N)$ indicates that the $j^{\text{th}}$ gene shows phenotype-specific activities. We then propose the following statistics by combining the regulatory effect and Jaccard distance to incorporate comprehensive information of gene networks:
    (7) $$Wd_{j}^{\left(t\right)}(C,N)=dJI_{j}\cdot d_{j}^{\left(t\right)}(C,N),\;t=1,2.$$
    The statistic $Wd_{j}^{\left(t\right)}(C,N)$ can be considered as the weighted version of $d_{j}^{\left(t\right)}(C,N)$ by the dissimilarity of the edge structure.
2. Enrichment score of the query gene network
  The enrichment score measures the degree to which $Wd_{j}^{\left(t\right)}(C,N)$ or $d_{j}^{\left(t\right)}(C,N)$ values of genes involved in a specific pathway (i.e. $V^{T}$) is over-represented at the top or bottom of the ordered values of $Wd_{i}^{\left(t\right)}(C,N)$ or $d_{i}^{\left(t\right)}(C,N)$ of the genes in the query gene network (i.e. *V*). We describe the enrichment score computation based on the statistic $Wd_{j}^{\left(t\right)}(C,N)$, referred to as GbNEAtRJ. The enrichment score based on $d_{j}^{\left(t\right)}(C,N)$, called GbNEAtR, can be computed by replacing $Wd_{j}^{\left(t\right)}(C,N)$ with $d_{j}^{\left(t\right)}(C,N)$.
  1. Rank the genes in the node set *V* of the query gene network and form the gene ranking list $L=\{g_{\left(1\right)},g_{\left(2\right)},\ldots ,g_{\left(p\right)}\}$ according to the ordered statistic of $Wd_{j}^{\left(t\right)}(C,N)$.
  2. Evaluate the fraction of genes involved in a specific pathway (i.e. $V^{T}$). The evaluation is based on the fraction of genes in $V^{T}(\prime \prime \text{hit}\prime \prime )$ weighted by their statistic $Wd_{j}^{\left(t\right)}(C,N)$ and the fraction of genes not in $V^{T}(¨\text{miss}")$ up to a given position *i* in *L*.
    (8) $$\begin{array}{cc} \text{Hit}(V^{T},i) & =\sum_{g_{\left(j\right)}\in V^{T},j\leq i}\frac{|Wd_{\left(j\right)}^{\left(t\right)}(C,N){|}^{k}}{V^{R}}, \end{array}$$
     
    (9) $$\text{where }V^{R}=\sum_{g_{\left(j\right)}\in V^{T}}|Wd_{\left(j\right)}^{\left(t\right)}(C,N){|}^{k},\text{Miss}(V^{T},i)=\sum_{g_{\left(j\right)}\notin V^{T},j\leq i}\frac{1}{\left(|V|-|V^{T}|\right)},$$
    where |V| is the size of node set of the query network (i.e. number of genes in the query network) and $\left|V^{T}\right|$ is a number of genes involved in the target pathway. The enrichment score *ES* is defined as the maximum deviation from the zero of $\text{Hit}(V^{T},i)-\text{Miss}(V^{T},i)\;\text{for}\;i\in V$. The *ES* increases if a gene ($g_{\left(j\right)}$) in the list *L* is in the gene set $V^{T}$ given as Equation (8) and decreases otherwise given in Equation (9). The *ES* can be interpreted as a weighted version of Kolmogorov–Smirnov-like statistic, which is a statistical test of “goodness of fit.” When k=0, the *ES* reduces to the standard (i.e. unweighted) Kolmogorov–Smirnov statistic; when k=1, we are weighting the genes in $V^{T}$ by the statistics $Wd_{j}^{\left(t\right)}$ normalized by the sum of the $Wd_{j}^{\left(t\right)}$ over all of the genes in $V^{T}$. The *k* is a tuning parameter giving higher weights to genes having large value of $Wd_{j}^{\left(t\right)}$ and can be selected by using a criterion. For the randomly distributed genes in $V^{T}$, $ES(V^{T})$ will be relatively small, while the value of $ES(V^{T})$ will be large if the genes in $V^{T}$ are concentrated at the top or bottom of the gene ranking list *L*.
3. Access significance of the enrichment score
  We assessed the significance of the enrichment score by comparing it with a set of permuted enrichment scores.
  1. Randomly assign the original phenotype labels *C* and *N* to cell lines, then estimate two gene networks based on permuted cell lines for phenotypes *C* and *N*, respectively.
  2. Recompute $ES(V^{T},\pi )$ for π=1,…,Π permutations and normalize $ES(V^{T})$ and $ES(V^{T},\pi )$, separately rescaling the positive and negative scores by dividing them by the mean of the $ES(V^{T},\pi )$ to compute the normalized enrichment scores $\mathrm{nES}(V^{T},\pi )$ and $\mathrm{nES}(V^{T})$
  3. Compute permutation *P*.value
    (10) $$p.\text{value}=\frac{\sum_{\pi =1}^{\Pi }I(\mathrm{nES}(V^{T},\pi ))\geq \mathrm{nES}(V^{T}))}{\Pi },$$
    where I(·) is an indicator function. The association between the query gene network with a target pathway is statistically significant when the *P*.value is smaller than the significance level α.
4. Multiple hypothesis testing
  For multiple hypothesis testing, we used the FDR-q value, i.e. the Benjamini–Hochberg correction (Benjamini and Hochberg 1995).

## 3 Results

### 3.1 Simulation studies

Simulation studies were conducted to demonstrate the performance of the developed strategies. For the target pathways, we use the KEGG pathway database (https://www.genome.jp/kegg/pathway.htm). We focused on the pathways related to human diseases, especially cancer (i.e. *cancer: specific types*), as shown in Table 1. The CCLE expression dataset consisting of mRNA expression levels of 19 221 genes for 1406 cells was used to estimate gene networks using the DepMap database (https://depmap.org/portal/). The CCLE dataset comprises various types of cancer cell lines, including colon/colorectal cancer (n=85), gastric cancer (n=48), non-small cell lung cancer (n=156), and small-cell lung cancer (n=77); non-cancerous cell lines (n=103) were also included. For each cancer in Table 1, we considered two phenotypes for cancer and non-cancerous cell lines, e.g. colorectal cancer and non-cancerous cell lines. To estimate the gene network involved in colorectal cancer pathway, we used the expression levels of 85 colon/colorectal cancer cell lines for the 87 genes involved in the pathway and randomly selected p−87 genes from those with the highest variance in expression levels (i.e. the gene network was estimated based on *p* genes and 85 colon/colorectal cancer cell lines). The lasso (i.e. δ=1 in Equation (2)) was used to estimate the gene networks, where the regularization parameter λ was selected by using the following Bayesian Information Criterion (Hui *et al.* 2007),


(11) $$\text{BIC}=\frac{||y_{\ell }-{\hat{y}}_{\ell }|{|}^{2}}{n\sigma^{2}}+\frac{\;\text{log}(n)}{n}\hat{df},$$

where $\hat{df}$ is the degree of freedom of the estimated model. We pick a relatively small values for λ, for example (0, 0.001, 0.01, 0.1, 0.5, 1). Then, for each value of λ, we compute the BIC and then choose the optimal regularization parameter minimizing the BIC.

**Table 1.** Cancer-specific types-related KEGG pathways.

Entry	Name	# Genes
hsa05210	Colorectal cancer	87
hsa05226	Gastric cancer	150
hsa05223	Non-small cell lung cancer	73
hsa05222	Small cell lung cancer	93

We also estimated the gene network for non-cancerous phenotypes based on the expression levels of *p* genes and 103 non-cancerous cell lines. For the gene networks of two phenotypes (i.e. colorectal cancer (C) and non-cancerous (N)), we applied the proposed strategy and computed the statistic $Wd_{j}^{\left(t\right)}(C,N)$ for j=1,…,p genes in the estimated network (i.e. j∈V). We ranked the *p* genes according to $Wd_{j}^{\left(t\right)}(C,N)$ and determined whether the 87 genes involved in the colorectal cancer pathway were over-represented in the leading subset of the gene ranking list of the *p* genes. We then computed the enrichment scores with k=0 based on Equations (8) and (9), and accessed the enrichment of the network in the colorectal cancer pathway. The result that the gene network was significantly enriched in the colorectal cancer pathway was considered a true positive for gene network-enriched pathway identification. For the true-negative scenario, we also estimated a gene network that was not involved in the colorectal cancer pathway. We consider a small portion (e.g. 1%, 5%, 10%) of genes involved in the colorectal cancer pathway and a large portion of genes that are not involved in the colorectal cancer pathway. In this study, we estimate gene network that is not enriched in colorectal cancer pathway based on the expression levels of the randomly selected 5% colorectal cancer genes and randomly selected p−5 genes from genes with the highest variance in expression levels. If the colorectal cancer pathway was identified as a significant pathway enriched in the network, we determined the result as a false positive. We considered the number of genes p=500,1000, and Π=200 permutations. The gene networks were estimated 50 times for true-positive and true-negative scenarios, that is, the simulations were performed based on 100 iterations. Similar processes were conducted for other cancer type-related KEGG pathways (i.e. gastric cancer, non-small cell lung cancer, and small cell lung cancer).

**Figure 2.**
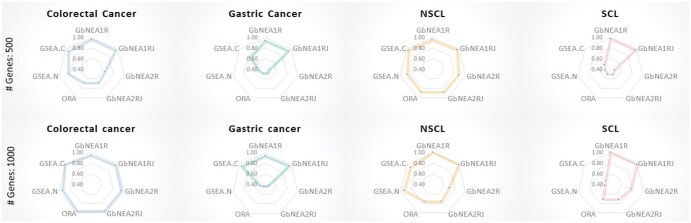
Accuracy of enriched pathway identification (GbNEA1R, GbNEA1RJ, GbNEA2R, and GbNEA2RJ, gene behaviors-based network enrichment analysis based on $d^{{\left(1\right)}_{j}}$, $Wd^{{\left(1\right)}_{j}}$, $d^{{\left(2\right)}_{j}}$ and $Wd^{{\left(2\right)}_{j}}$, respectively; GSEA.N, gene set enrichment analysis with network; GSEA.C, gene set enrichment analysis with centrality measure).

We evaluated our strategies by comparing them with existing strategies, that is, ORA, gene set enrichment analysis with network (GSEA.N), and gene set enrichment analysis with centrality measure (GSEA.C), where the node set of the gene network is considered a gene set (Jung 2024). For the estimated gene networks of each cancer and non-cancerous (i.e. non-permuted networks), the existing methods (i.e. ORA, GSEA.N, and GSEA.C) were applied, where the t-statistic of difference of expression levels of genes between cancer and non-cancerous samples is used as a gene-level statistic. These methods were implemented using the R package *gsean* (Jung 2024). The evaluation was based on the Accuracy, Precision, F1 score, Recall, and True-negative rate. Figure 2 shows the accuracy of the gene network-enriched pathway identification. As shown in Fig. 2, the proposed strategies, GbNEA1R, and GbNEA1RJ show outstanding performances compared with the other methods, especially the comprehensive information-based strategy (GbNEA1RJ) exhibited the best overall performance. Although GSEN.C, GbNEA2R, and GbNEA2RJ also showed effective results, the methods did not show stably effective performances, i.e. the methods show poor results in gene network enrichment analysis for some cancer-related pathways.

Table 2 lists the evaluation results based on the various performance measures. It can also be observed from Table 2 that GbNEA1R and GbNEA1RJ show effective results from the viewpoint of various performance measures. ORAm GbNEA2R and GbNEA2RJ provide poor true-negative rate results in gene network-enriched pathway identification for some cancers.

**Table 2.** Results of gene network-enriched analysis, where “–” indicates that precision cannot be computed because there are no selected pathways.

Cancers	Methods	# Genes: 500					# Genes: 1000				
		Accuracy	Precision	Recall	F1.score	TNR	Accuracy	Precision	Recall	F1.score	TNR
Colorectal cancer	GbNEA1R	**0.96**	1.00	0.92	0.96	1.00	0.94	0.94	0.94	0.94	0.94
	GbNEA1RJ	**0.96**	1.00	0.92	0.96	1.00	0.96	0.96	0.96	0.96	0.96
	GbNEA2R	0.96	1.00	0.92	0.96	1.00	0.65	0.80	0.40	0.53	0.90
	GbNEA2RJ	0.96	1.00	0.92	0.96	1.00	0.69	0.91	0.42	0.58	0.96
	ORA	0.77	0.68	1.00	0.81	0.54	0.50	0.50	1.00	0.67	0.00
	GSEA.N	0.83	1.00	0.66	0.80	1.00	0.93	1.00	0.86	0.92	1.00
	GSEA.C	0.93	1.00	0.86	0.92	1.00	**1.00**	1.00	1.00	1.00	1.00
Gastric cancer	GbNEA1R	0.93	0.88	1.00	0.93	0.86	0.94	0.89	1.00	0.94	0.88
	GbNEA1RJ	**0.94**	0.89	1.00	0.94	0.88	**0.95**	0.91	1.00	0.95	0.90
	GbNEA2R	0.47	0.20	0.02	0.04	0.92	0.48	0.33	0.04	0.07	0.92
	GbNEA2RJ	0.44	0.00	0.00	0.00	0.88	0.49	0.44	0.08	0.14	0.90
	ORA	0.86	0.78	1.00	0.88	0.72	0.52	0.51	1.00	0.68	0.04
	GSEA.N	0.52	1.00	0.04	0.08	1.00	0.50	–	0.00	0.00	1.00
	GSEA.C	0.75	1.00	0.50	0.67	1.00	0.93	1.00	0.86	0.92	1.00
NSCL	GbNEA1R	0.95	0.91	1.00	0.95	0.90	**1.00**	1.00	1.00	1.00	1.00
	GbNEA1RJ	**0.97**	0.94	1.00	0.97	0.94	**1.00**	1.00	1.00	1.00	1.00
	GbNEA2R	0.70	0.71	0.68	0.69	0.72	0.89	0.82	1.00	0.90	0.78
	GbNEA2RJ	0.76	0.73	0.82	0.77	0.70	0.88	0.81	1.00	0.89	0.76
	ORA	0.95	0.91	1.00	0.95	0.90	**1.00**	1.00	1.00	1.00	1.00
	GSEA.N	0.87	0.85	0.90	0.87	0.84	0.93	0.89	0.98	0.93	0.88
	GSEA.C	0.95	0.91	1.00	0.95	0.90	0.91	0.85	1.00	0.92	0.82
SCL	GbNEA1R	**0.97**	0.94	1.00	0.97	0.94	**1.00**	1.00	1.00	1.00	1.00
	GbNEA1RJ	**0.97**	0.94	1.00	0.97	0.94	**1.00**	1.00	1.00	1.00	1.00
	GbNEA2R	0.78	0.97	0.58	0.73	0.98	0.48	0.47	0.28	0.35	0.68
	GbNEA2RJ	0.72	0.96	0.46	0.62	0.98	0.51	0.52	0.34	0.41	0.68
	ORA	0.50	0.50	1.00	0.67	0.00	0.50	0.50	1.00	0.67	0.00
	GSEA.N	0.50	–	0.00	0.00	1.00	0.50	–	0.00	0.00	1.00
	GSEA.C	0.53	1.00	0.06	0.11	1.00	0.51	1.00	0.02	0.04	1.00
Average	GbNEA1R	0.95	0.93	**0.98**	0.95	0.93	0.97	0.96	0.99	0.97	0.96
	GbNEA1RJ	**0.96**	0.94	**0.98**	**0.96**	0.94	**0.98**	**0.97**	0.99	**0.98**	**0.97**
	GB-NEA.R2	0.73	0.72	0.55	0.60	0.91	0.63	0.60	0.43	0.46	0.82
	GB-NEA.R2J	0.72	0.67	0.55	0.59	0.89	0.64	0.67	0.46	0.50	0.83
	ORA	0.77	0.72	1.00	0.83	0.54	0.63	0.63	**1.00**	0.75	0.26
	GSEA.N	0.68	0.95	0.40	0.44	0.96	0.72	0.95	0.46	0.46	**0.97**
	GSEA.C	0.79	**0.98**	0.61	0.66	**0.97**	0.84	0.96	0.72	0.72	0.96

Bold numbers indicate the best performance among the methods.

Table 3 shows the running times of the gene networks enrichment analysis by using GbNEA (i.e. GbNEA1RJ), ORA, GSEA.N, and GSEA.C, where ORA, GSEA.N, and GSEA.C are implemented by using the R package *gsean*.

**Table 3.** Running time (in seconds) of gene network enrichment analysis by using GbNEA, ORA, GSEA.N, and GSEA.C.

		Colorectal cancer	Gastric cancer	NSCL	SCL
500	GbNEA	3.9	3.0	4.5	3.3
	ORA	51.0	41.5	41.7	43.8
	GSEA.N	0.8	0.7	0.7	0.7
	GSEA.C	3.2	0.5	0.6	0.6
1000	GbNEA	18.5	13.5	24.1	14.8
	ORA	49.8	94.1	85.5	71.5
	GSEA.N	1.1	1.0	1.4	1.1
	GSEA.C	0.9	0.9	0.9	0.9

As shown in Table 3, our strategy does not demonstrate competitive performance in terms of computational complexity when compared to GSEA.N and GSEA.C, which exhibit the highest computational efficiency. On the other hand, ORA suffers from computational complexity. However, the runtime of each approach remains within an acceptable range and does not hinder the applicability of the methods.

### 3.2 Uncovering immune disease pathways enriched with the COVID-19 severity-specific gene networks

The immune system is essential in viral infections, including those caused by coronaviruses, by activating antiviral defense mechanisms and influencing the progression, severity, and clinical outcomes of COVID-19. Although many studies have been conducted to uncover biomarkers related to immune system of COVID-19 severity, the existing studies were based on abnormalities in a single gene; thus, a comprehensive analysis of the molecular perturbation was not performed. However, the complex mechanism of COVID-19 severity involves numerous perturbed genes in the molecular network.

We aimed to uncover COVID-19 severity (i.e. asymptomatic, mild, severe, most severe)-specific immune disease pathways and analyzed whole-blood RNA-seq data of 1102 genotyped samples obtained from the Japan COVID-19 Task Force (Wang *et al.* 2022a). The Japan COVID-19 Task Force defined the four levels of COVID-19 severity as “asymptomatic” for patients without COVID-19 related symptoms, “Mild” for other symptomatic patients, “Severe” for others requiring oxygen support, and “Most severe” for patients in intensive care or requiring intubation and ventilation (Wang *et al.* 2022a).

To identify the COVID-19 severity-specific immune disease pathways, we consider “*Immune disease*” pathways in the KEGG pathway database as shown in Table 4. For asymptomatic (*n* = 71), mild (*n* = 241), severe (*n* = 404), and most severe (*n* = 303) labeled samples, we extracted the expression levels of the 332 immune disease genes, where 294 genes having expression levels in more than 10% of samples are used to estimate gene networks. From the genes not involved in “*Immune disease*” pathways, we also extracted 294 genes with the highest variance of expression levels in all samples. For the 588 genes, we then estimated four immune disease genes networks for the asymptomatic (level 1), mild (level 2), severe (level 3), and most severe (level 4) samples (i.e. immNW.Lv1, immNW.Lv2, immNW.Lv3, immNW.Lv4) by using the lasso. We also estimated immune disease gene networks, immNW.Lv234, immNW.Lv134, immNW.Lv124, and immNW.Lv123, which were estimated by the expression levels of the 588 immune disease genes for levels 2,3,4, levels 1,3,4, levels 1,2,4, and levels 1,2,3 samples, respectively. That is, we consider samples for level 1 (2, 3, 4) and samples for level “234” (“134,” “124,” “123”) as two phenotypes, and compute the statistic $Wd_{j}^{\left(1\right)}$(Lv1, Lv234) ($Wd_{j}^{\left(1\right)}$(Lv2, Lv134), $Wd_{j}^{\left(1\right)}$(Lv3, Lv124), and $Wd_{j}^{\left(1\right)}$(Lv4, Lv123)). The main goal of the analysis was to identify immune disease pathways enriched with the gene network immNW.Lv1 (immNW.Lv2, immNW.Lv3, and immNW.Lv4), which showed different interplays with immNW.Lv234 (immNW.Lv134, immNW. Lv124, and immNW.Lv123). To achieve this goal, we performed the proposed gene network enrichment analysis (GbNEA1RJ with k=0) and identified immune disease pathways enriched with gene networks at a particular COVID-19 severity level.

**Table 4.** Immune disease-related KEGG pathways.

Entry	Name	# Genes
hsa05310	Asthma	32
hsa05322	Systemic lupus erythematosus	141
hsa05323	Rheumatoid arthritis	95
hsa05320	Autoimmune thyroid disease	54
hsa05321	Inflammatory bowel disease	66
hsa05330	Allograft rejection	39
hsa05332	Graft-versus-host disease	45
hsa05340	Primary immunodeficiency	38
Total	Immune disease	332

**Figure 3.**
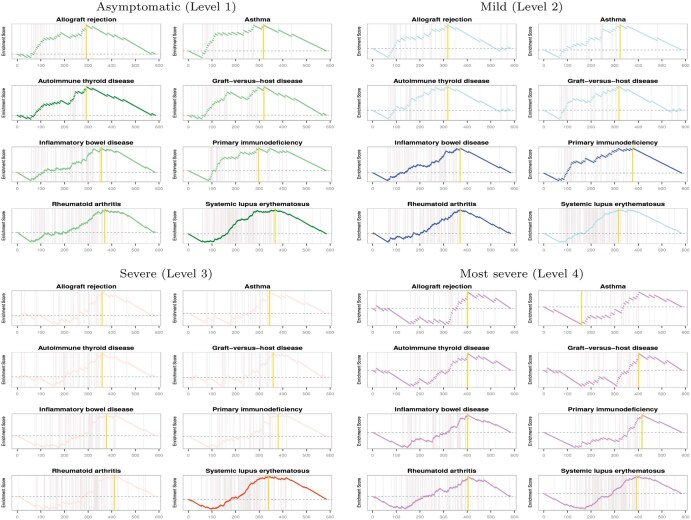
Enrichment score behaviors of immune disease-related pathways for gene networks of COVID-19 of “Asymptomatic (level 1),” “Mild (level 2),” “Severe (level 3),” and “Most severe (level 4)” samples, where yellow vertical lines indicate the locations of the maximum deviation from zero, i.e. the enrichment scores.

Table 5 shows the results of the COVID-19 severity-specific gene network enrichment analysis (i.e. *P*-values). As shown in Table 5, the gene networks for asymptomatic (level 1) samples were enriched in the “Autoimmune thyroid disease” and “Systemic lupus erythematosus.” The network of mild (level 2) samples was involved in various immune disease pathways, such as “Inflammatory bowel disease,” “Primary immunodeficiency,” and “Rheumatoid arthritis.” The “Systemic lupus erythematosus” was also identified for gene networks of severe (level 3) samples. One of the surprising and interesting results was that there were no significantly enriched pathways for the most severe (level 4) sample-specific molecular interplay. The network of most severe samples (level 4) has relatively smaller enrichment scores ($\mathrm{nES}(V^{T})$) for the eight immune disease pathways than gene networks of other COVID-19 levels, while its permuted scores ($\mathrm{nES}(V^{T},\pi )$) were similar to those of network of levels 1, 2, and 3. Thus, there was no significant immune disease pathways. The findings indicate that the genes involved in immune disease pathways do not show characteristic molecular interactions in most severe samples of COVID-19. Figure 3 shows the behaviors of the enrichment scores of the COVID-19 severity-specific gene networks for the identified immune disease pathways. The genes involved in the identified immune disease pathways (i.e. “Autoimmune thyroid disease,” “Inflammatory bowel disease,” “Primary immunodeficiency,” “Rheumatoid arthritis,” and “Systemic lupus erythematosus”) were over-represented at the bottom of the gene ranking list from the gene networks in overall.

**Table 5.** Significance of immune disease pathways for gene networks of COVID-19 severity levels (i.e. *P*.value).

	Level 1	Level 2	Level 3	Level 4
Allograft rejection	0.114	0.572	0.109	0.597
Asthma	0.547	0.577	0.154	0.985
Autoimmune thyroid disease	0.000	0.592	0.065	0.876
Graft-versus-host disease	0.502	0.448	0.154	0.522
Inflammatory bowel disease	0.169	0.000	0.169	0.985
Primary immunodeficiency	0.080	0.000	0.333	0.995
Rheumatoid arthritis	0.308	0.000	0.284	0.881
Systemic lupus erythematosus	0.000	0.065	0.020	0.995

- Autoimmune thyroid disease
  COVID-19 survivors exhibited a 2-fold increase in autoimmune thyroid disease prevalence relative to matched controls by age and sex, implying a potential link between SARS-CoV-2 infection and thyroid autoimmunity (Rossini *et al.* 2023). Established links between COVID-19, cytokine release syndrome, and autoimmunity support the hypothesis that COVID-19 may contribute to the onset of autoimmune thyroid disorders, including autoimmune hypothyroidism (Naguib 2022).
- Rheumatoid arthritis
  Patients with rheumatoid arthritis face a significantly higher risk of COVID-19 infection and severe outcomes, and evidence suggests that COVID-19 may also increase the incidence of inflammatory arthritis (Marín *et al.* 2023, Jin *et al.* 2024).
- Systemic lupus erythematosus
  Systemic lupus erythematosus (SLE) is an autoimmune disease characterized by the immune system’s misdirected response, leading to harm to normal tissues. Nian *et al.* (2024) suggested that the interplay between SLE and COVID-19 drives the activation of the JAK-STAT signaling pathway triggered by IFN-I/II in monocytes/macrophages. Wang *et al.* (2022b) discovered that the genome-wide genetic association signal for severe COVID-19 is linked to that of SLE.
- Inflammatory bowel disease
  Inflammatory bowel disease (IBD) is a long-lasting systemic inflammatory condition that mainly affects the gastrointestinal system, encompassing both Crohn’s disease and ulcerative colitis (Iqbal *et al.* 2024). It is suggested that patients with IBD could face more severe complications from COVID-19 (Iqbal *et al.* 2024). Iqbal *et al.* (2024) revealed that hospitalized COVID-19 patients with a history of IBD had a greater risk of complications such as sepsis, shock, and acute kidney injury.
- Primary immunodeficiency
  Primary immunodeficiency (PID) disorders represent a heterogeneous group of genetic conditions that severely impair immune function (Peddi *et al.* 2023). Gray *et al.* (2022) discovered that differences in individual susceptibility to severe COVID-19 may be due to an increased number of otherwise healthy individuals with undiagnosed asymptomatic PID, which is revealed by SARS-CoV-2 infection. Adults with PID and symptomatic SID face increased morbidity and mortality from COVID-19 when compared to those of the general population (Shields *et al.* 2021).

**Figure 4.**
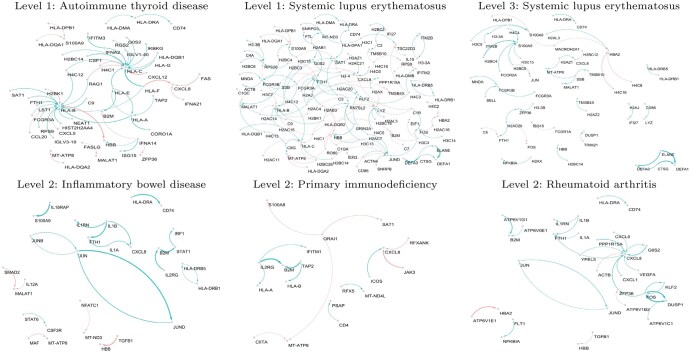
Molecular interplays of COVID-19 severity levels involved in significantly enriched immune disease pathways. Edge thickness represents the edge weight from *X* to *Y* genes estimated by the lasso, i.e. ${\hat{\beta }}_{XY}$, color indicates sign of the edge (red and blue are “+” and “−,” respectively), and arrow (X → Y) indicates that gene X regulates gene Y.

For effective visualization, we considered only the 1% largest absolute edge weights and visualized the interplay between the genes involved in each pathway. As shown in Fig. 4, COVID-19 asymptomatic samples (level 1) have relatively higher activities of molecular interplay than those of mild and severe samples; that is, the network of level 1 samples consists of a large number of genes and edges. Especially, the molecular interplays of mild samples show sparse networks.

In the gene networks associated with the identified immune disease pathways, HLA class I (i.e. HLA-A, HLA-B, HLA-C) are main players in molecular interplays associated with immune disease pathways. Vanaga *et al.* (2023) revealed that the association of HLA class II alleles with epithelial cell apoptosis and extracellular matrix production indirectly supports the involvement of HLA genes in acute COVID-19 severity. Additionally, this regional study provided evidence that the HLA genotype affects the clinical outcomes of COVID-19 (Langton *et al.* 2021). It can also be seen in Fig. 4 that CXCL8 and S100A9 were hub genes in the COVID-19 severity-specific gene networks. The hub genes have been revealed as biomarkers of COVID-19 in the previous studies (Guo *et al.* 2021, Khalil *et al.* 2021, Mellett and Khader 2022, Cambier *et al.* 2023). Previous studies strongly support our findings that the identified genes and their molecular interplays are crucial markers for understanding the immune disease mechanism of COVID-19.

## 4 Discussion

We introduced novel computational strategies called GbNEA to uncover functional pathways enriched with phenotype-specific gene networks. The proposed strategy measures the enrichment of gene networks based on comprehensive gene network information (i.e. gene expression levels, edge weight, and structure). Thus, we could effectively identify pathways enriched in the query network, leading to biologically interpretable results.

Simulation studies were conducted to illustrate the performance of the proposed strategy. For the four types of cancer, we estimated gene networks for cancer and non-cancerous cell lines and then performed enriched pathway identification. Simulation results demonstrated that our strategy provide, especially GbNEA1RJ, outstanding performance for phenotype-specific gene network enrichment analysis.

We applied the proposed GbNEA to uncover the immune disease pathways enriched with COVID-19 severity-specific gene networks in Japanese population. Our results uncovered that gene networks of COVID-19 asymptomatic and sever samples involved in the “Systemic lupus erythematosus” pathway, while that of mild samples were associated with “Inflammatory bowel disease,” “Primary immunodeficiency pathways,” and “Rheumatoid arthritis.” The COVID-19 severity-specific gene network pathway analysis also revealed CXCL8 and S100A9 as crucial hub genes, and HLA class I (i.e. HLA-A, HLA-B, HLA-C) as the main player in the gene networks involved in immune disease pathways; these results have been validated in the literature. Collectively, we suggest that controlling CXCL8 and S100A9 and the main players HLA class I is crucial to uncover the immune disease-related mechanisms of COVID-19 severity. A noteworthy and unexpected finding was the absence of significantly enriched pathways in the molecular interplay specific to the most severe (level 4) samples. The results suggest that genes involved in immune disease pathways do not exhibit distinctive molecular interactions in the most severe COVID-19 samples. It can be considered through the results that the higher stage of COVID-19 cannot be explained by only immune disease pathways, but rather involves more complex biological mechanisms. The enrichment analysis for the gene network of most sever COVID-19 samples based on not only immune disease but also various diseases remains the topic of future work of the current study.

Although our method yielded effective results in the functional enrichment analysis of gene networks, the current study has several limitations. *Computational complexity*: As demonstrated in the simulation study, our strategy is less computationally efficient than existing methods. This remains a challenge that must be addressed to enable the analysis of larger-scale networks. *Biological validation*: Although the immune disease pathways identified from COVID-19 severity-related gene networks were supported by prior studies, experimental validation would provide further confirmation and highlight the practical value of our proposed approach. *High reliance on gene network estimation*: Our strategy relies on gene networks estimated through computational methods. Hence, robust and effective network inference is critical for meaningful enrichment results.

Our strategy facilitates the systematic interpretation of large-scale gene networks and the identification of disease-associated biological pathways, thereby enabling a comprehensive understanding of the molecular mechanisms underlying complex diseases. While the current study applied enrichment analysis to explore COVID-19 severity-related gene networks, the simulation results suggest that the proposed GbNEA is also effective in identifying cancer-associated pathways, highlighting its potential utility in research on various diseases, e.g. cancer research.

## Data Availability

RNA-seq data from the Japan COVID-19 Task Force are available from the National Bioscience Database Center Human Database (accession code: hum0343; https://humandbs.biosciencedbc.jp/en/hum0343).
